# Periodontal Disease and Adverse Neonatal Outcomes: A Systematic Review and Meta-Analysis

**DOI:** 10.3389/fped.2022.799740

**Published:** 2022-05-04

**Authors:** Youzhen Zhang, Wanbing Feng, Jingyu Li, Linlin Cui, Zi-Jiang Chen

**Affiliations:** ^1^Center for Reproductive Medicine, Cheeloo College of Medicine, Shandong University, Jinan, China; ^2^Key Laboratory of Reproductive Endocrinology of Ministry of Education, Shandong University, Jinan, China; ^3^Shandong Key Laboratory of Reproductive Medicine, Jinan, China; ^4^Shandong Provincial Clinical Research Center for Reproductive Health, Jinan, China; ^5^National Research Center for Assisted Reproductive Technology and Reproductive Genetics, Shandong University, Jinan, China; ^6^Shanghai Key Laboratory for Assisted Reproduction and Reproductive Genetics, Shanghai, China; ^7^Center for Reproductive Medicine, Ren Ji Hospital, School of Medicine, Shanghai Jiao Tong University, Shanghai, China

**Keywords:** periodontal disease (PD), neonatal outcomes, preterm birth (PTB), low birth weight (LBW), meta-analysis

## Abstract

**Objective:**

The aim of this study was to evaluate the association between maternal periodontal disease (PD) and three main adverse neonatal outcomes, namely, preterm birth (PTB), low birth weight (LBW), and small for gestational age (SGA).

**Methods:**

The Ovid Medline, Web of Science, Embase, and Cochrane Library were searched up to 6 December 2020 for relevant observational studies on an association between PD and risk of PTB, LBW, and SGA. Eligibility criteria included observational studies which compared the prevalence of PTB and/or LBW and/or SGA between PD women and periodontal health controls. The exclusion criteria included incomplete data, animal research, and mixing up various pregnancy outcomes, such as “preterm low birth weight” and languages other than Chinese and English. Data were extracted and analyzed independently by two authors. The meta-analysis was performed using Stata Statistical Software, Release 12 (StataCorp LP, College Station, TX, USA). Odds ratio (OR), confidence intervals (CIs), and heterogeneity (*I*^2^) were computed.

**Results:**

Fourteen case-control studies and 10 prospective cohort studies, involving 15,278 participants, were identified. Based on fixed effect meta-analysis, PTB showed a significant association with PD (OR = 1.57, 95% CI: 1.39–1.77, *P* < 0.00001) and LBW also showed a significant association with PD (OR = 2.43, 95% CI: 1.75–3.37, *P* < 0.00001) in a random effect meta-analysis. However, a random effect meta-analysis showed no relationship between PD and SGA (OR = 1.62, 95% CI: 0.86–3.07, *P* = 0.136).

**Conclusion:**

Our findings indicate that pregnant women with PD have a significantly higher risk of PTB and LBW. However, large prospective, blinded cohort studies with standardized diagnostic criteria of PD and adequate control of confounding factors are still required to confirm the relationship between PD and adverse neonatal outcomes.

## Introduction

Preterm birth (PTB), low birth weight (LBW), and small for gestational age (SGA) are the leading adverse neonatal outcomes worldwide and have significant public health implications because they are responsible for a great part of neonatal mortality and morbidity in both developed and developing countries (1–3). According to the World Health Organization (WHO), PTB is defined as a delivery that takes place before 37 weeks (<259 days) of gestation, LBW refers to birth weight of <2,500 g, and SGA refers to birth weight below the 10th percentile of birth weight for gestational age (3, 4). Convincing evidence has found the association between adverse neonatal outcomes and infections especially genitourinary infections (5, 6). However, the hypothesis that infections distant from the feto-placental unit may be associated with adverse neonatal outcomes has led to an increased awareness of the potential role of chronic bacterial infections elsewhere in the body (7).

Periodontal disease (PD) occurs in ~40% of pregnant women (8). It includes several inflammatory conditions, usually initiated by oral bacteria, starting with a reversible build-up of plaque and inflammation of gingival tissue (gingivitis), progressing to irreversible destruction of the supportive periodontal tissues of the teeth and tooth loss (periodontitis) (9). Generally, PD is clinically characterized by periodontal pocket depth (PPD), clinical attachment level (CAL), alveolar bone loss, and gingival inflammation (measured as bleeding on probing) (10). In 1996, Offenbacher et al. conducted a case-control study (11), suggesting that maternal PD could lead to a 7-fold increase in the risk of preterm LBW (PLBW). Following this groundbreaking study, numerous studies have shown an association between periodontal inflammation and adverse neonatal outcomes, including PTB, LBW, and SGA (12, 13). However, this association has not been consistent in other studies (14–17). These inconsistencies could be explained by several factors as follows: (i) lack of a unified diagnostic standard for PD (18); (ii) the variety of definitions used for adverse pregnancy outcomes (APOs), such as PLBW, preterm or LBW, and preterm and/or LBW; (iii) confounding effect of the risk factors. There have been a few systematic reviews and meta-analyses on the relationship between PD and adverse neonatal outcomes so far (19, 20). However, the heterogeneity of the previous studies still needs further exploration of confounding factors and more detailed subgroup analysis, and due to the publication of new data, it is necessary to perform a meta-analysis which can improve the evidence on the association between PD and adverse neonatal outcomes.

Given the alarming global disease burden of PD and adverse neonatal outcomes, there is an urgent need to clarify the substantial role of PD in the etiology of adverse neonatal outcomes (21), which will provide evidence for the periodontal prevention and intervention among children-bearing women, thus reducing the incidence of adverse neonatal outcomes caused by PD. Therefore, the objectives of this systematic review and meta-analysis were to evaluate the association between PD and adverse neonatal outcomes and provide suggestions for preventive medicine and public health.

## Materials and Methods

This systematic review and meta-analysis were executed and reported according to the Preferred Reporting Item for Systematic Reviews and Meta-analysis statement (PRISMA) (22). An a priori protocol was written and followed. The Population/Income/Comparison/Outcome (PICO) question was set up as follows: Whether there was a higher risk of preterm birth and/or LBW and/or SGA in the population of pregnant women with periodontal disease compared with the population of periodontal health pregnant women.

### Information Sources and Search Strategy

The comprehensive database searches were performed by W. F. from inception to December 2020 in the following electronic sources: Ovid Medline 1946, Web of Science 1900, Embase 1947, and Cochrane Library. The following terms were used in the automatic search: “periodontal disease AND preterm delivery,” “periodontitis AND preterm delivery,” “periodontal disease AND low birth weight,” “periodontitis AND low birth weight,” “periodontal disease AND small for gestational age,” and “periodontitis AND small for gestational age.” The detailed search strategy can be found in Supplementary Table 1. A manual search of reference lists of relevant articles was also conducted. All the searched literature was exported to the reference manager software (ENDNOTE®X9, Bld 7212, Thomson Reuters), where the duplicated articles were removed. Manual checks and assessments were also performed to determine whether abstracts were unique or copies.

### Study Selection and Eligibility Criteria

Two independent reviewers Y. Z. and L. C. performed the selection of articles. The first screening was carried out by reading the title and the abstract, eliminating those studies that did not meet the predetermined eligibility criteria. Subsequently, intensive reading of the full text of the remaining articles was made, finally selecting those eligible articles. Inclusion criteria were as follows: (1) observational studies, including case-control studies and prospective cohort studies; (2) compared the prevalence of PTB and/or LBW and/or SGA between PD women and periodontal health controls; (3) dichotomous data were reported or sufficient data were available to calculate the odds ratio (OR) and its 95% confidence interval (CI); and (4) when overlapped studies appeared, choose the latest and most complete one. The exclusion criteria included the following: (1) incomplete data: unclear or inappropriate definition of cases, unadjusted confounders, and unavailable data; (2) animal research; (3) PTB or LBW or SGA were not used as independent observational outcomes separately, such as PLBW, preterm or LBW, and preterm and/or LBW; and (4) languages other than Chinese and English.

### Data Extraction and Quality Assessment

The full text of eligible studies was independently reviewed by Y. Z. and L. C. Meta-analysis was performed to determine whether maternal PD had an adverse effect on PTB, LBW, and SGA separately. Data extraction was also independently performed by the two reviewers. Extracted information of all eligible studies included title, author names, year of publication, country, study design, sample size, age (average and/or range), timing of measurements, definitions of cases, primary outcomes (prevalence of PTB/LBW/SGA expressed by the number of cases, calculated OR and its 95% CI), and adjusted confounders.

The methodological quality of eligible studies was evaluated using the Newcastle–Ottawa Quality Assessment Scale (NOS) (23), a standardized tool recommended by the Cochrane Working Group to assess the risk of bias in observational studies. Criteria for qualitative assessment comprised three main items, namely, (1) selection of sample, (2) comparability, and (3) exposure. Each item had questions with options and could receive one or two points if the criterion was achieved. Studies were graded into low quality (0–6 points) and high quality (7–9 points) by two independent reviewers (Y. Z. and L. C.). The reviewers resolved discrepancies by discussion and additional comments from a non-author investigator.

### Data Synthesis and Analysis

All analyses were performed using Stata (version 12, StataCorp, College Station, TX), as the *P*-value < 0.05 was considered to be statistically significant. *Q* test and *I*^2^ test were used to assess the heterogeneity across studies (24). The hazard ratio was considered equivalent to OR, and to estimate pregnancy outcomes in women with PD vs. periodontal healthy controls, a fixed or random-effect model was used to calculate the pooled OR. Subgroup analysis was performed to find whether particular characteristics of studies (clinical or methodological) were associated to the value of the overall OR. Publication bias was assessed graphically and statistically *via* the Egger's linear regression test at *P* < 0.10 (25). Finally, with the metaninf algorithm in Stata, sensitivity analysis was performed by excluding one study at each turn.

## Results

### Study Selection and Characteristics

**Figure 1:**
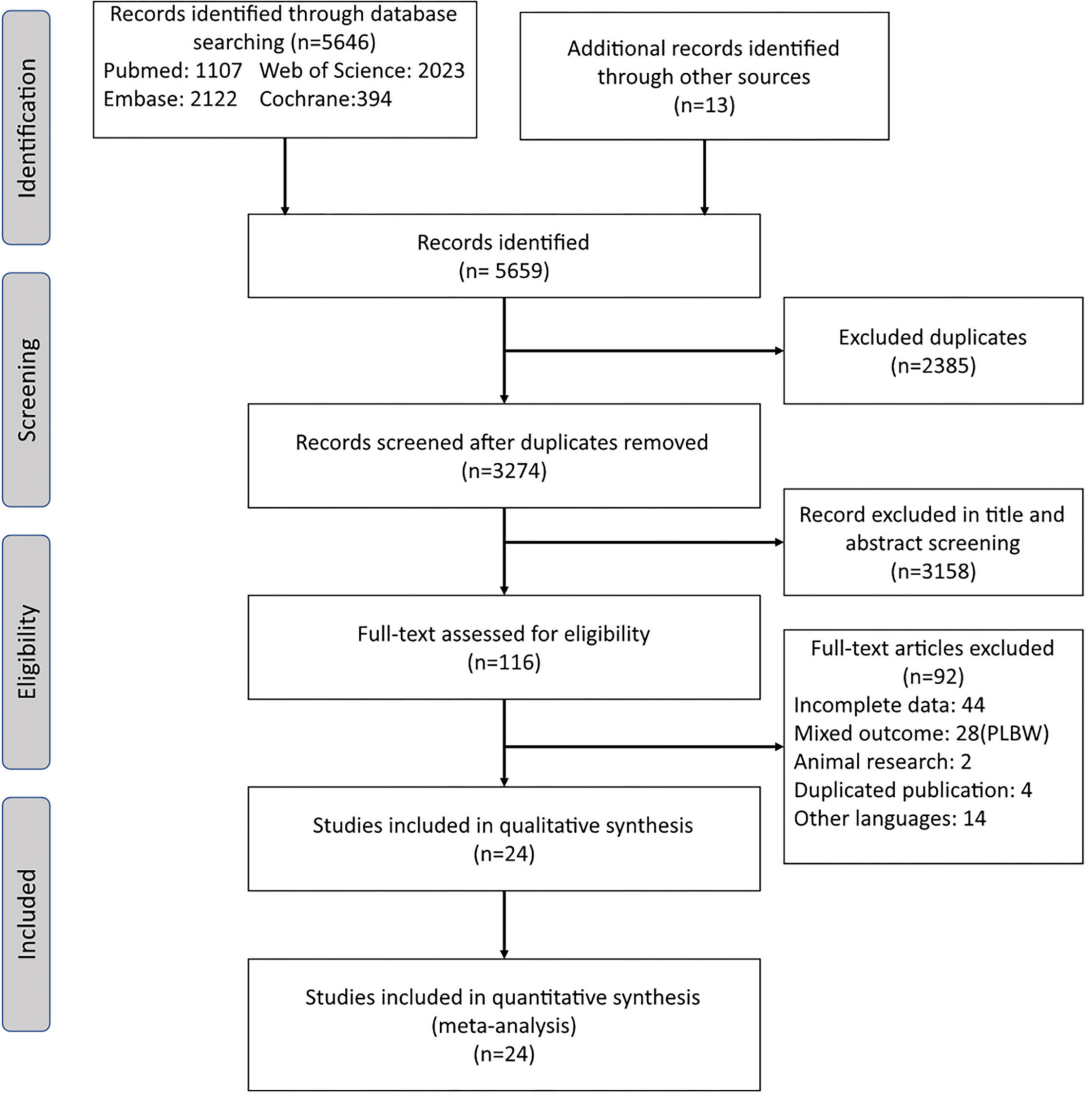
Flowchart of the study selection process for the present meta-analysis.

Search results are presented in the flowchart (Figure 1). A total of 5,646 articles were yielded in the electronic search, and 13 articles were searched from reference lists of relevant articles, reviews, and dissertations. After title and abstract screening, 116 articles underwent full-text assessment. Subsequently, 44 studies were excluded as a result of incomplete data, 28 were excluded for mixed outcomes, 2 were excluded for animal research, 4 studies were excluded for duplicated publication, and 14 studies were excluded for other languages. Finally, 24 observational studies were included in this meta-analysis (26–49).

The 24 eligible studies contained 10 prospective cohort studies and 14 case-control studies, including 15,278 pregnant women. The included studies were all women of childbearing age, almost all women were older than 18. All the studies compared the prevalence of APOs between PD women and periodontally healthy controls. Among them, 15 studies investigated PTB, 14 studies investigated LBW, and 4 studies investigated SGA. Definitions of PTB, LBW, and SGA were in accordance with the WHO standard. However, definitions of PD mainly came from previous epidemiological studies and were inconsistent, including definition from Lopez et al. (≥4 teeth with ≥1 site with PPD ≥4 mm and CAL ≥3 mm at the same site), definition from Offenbacher et al. (PD ≥3 mm or CAL ≥2 mm), definition from American Centers for Disease Control and Prevention (CDC) and American Academy of Periodontology (AAP) (CDC-APP, ≥ 2 interproximal sites with CAL ≥4 mm), and other. Characteristics of all studies are displayed in Table 1.

**Table 1:** Characters of included studies.

**Study**	**Country**	**Design**	**Match**	**Blind**	**No. of Participants**			**Age**		**Examination time**	**Definition of periodontal disease**	**Outcomes**	**OR (95% CI)**	**Conclusion**
					**All**	**PD**	**Control**	**PD**	**Control**					
Agueda et al. (26)	Spain	Prospective cohort	/	/	1,296	338	958	18–40^b^		About 20 weeks gestation	≥4 teeth with ≥1 site with PPD ≥4 mm and CAL ≥3 mm at the same site (Lopez)	PTB LBW	1.77 (1.08–2.88)^†^ /	No relationship was found between PTB, LBW and mother's PD
Baskaradoss et al. (27)	India	Case-control	No	NA	300	54	246	25.51 ± 3.01^a^		Within 48 h after delivery	≥4 teeth with ≥1 site with PPD ≥4 mm and CAL ≥3 mm at the same site (Lopez)	PTB	2.72 (1.68–6.84)^†^	Periodontal disease is a possible risk factor for PTB in this population
Bassini et al. (28)	Brazil	Case-control	Yes	No	915	511	404	NA		After delivery	≥3 sites, in different teeth, with CAL ≥3 mm	LBW	0.93 (0.63–1.41)^†^	Theres's no statistically significant association between PD and LBW
Boggess et al. (29)	USA	Prospective cohort	/	/	1,017	733	284	≥18^b^		1st or 2nd prenatal visit	≥1 tooth sites with PPD >4 mm or ≥1 tooth PPD >3 mm with BOP (WHO)	SGA	Mild: 1.3 (0.7–2.5)^†^ > Mild: 2.3 (1.1–4.5)	PD early in pregnancy is associated with delivery of a SGA infant
Cruz et al. (30)	Brazil	Case-control	No	Yes	302	137	165	NA		Within 7 days after delivery	≥4 teeth with ≥1 site with PPD ≥4 mm and CAL ≥3 mm at the same site (Lopez)	LBW	2.15 (1.32–3.48)*	PD is a possible risk factor for LBW
Erchick et al. (31)	Nepal	Prospective cohort	/	/	1,394	554	840	23.0 ± 4.6^a^		<26 weeks of GA	BOP ≥10% and/or PD ≥4 mm	PTB	NA	GS in women examined early in pregnancy were risk factors for PTB
Filho et al. (32)	Brazil	Case-control	No	Yes	372	72	300	23.86 ± 6.6^a^		Within 7 days after delivery	≥4 teeth with ≥1 site with PD ≥4 mm, CAL ≥3 mm, and BOP at the same site	LBW	6.02 (2.47–15.17)^†^	PS associated with LBW
Jacob et al. (33)	India	Case-control	Yes	Yes	340	137	203	18–35^b^		Within 48 h after delivery	≥1 site PPD ≥4 mm (WHO)	LBW	2.85 (1.62–5.50)^†^	PS is a significant risk factor for LBW
Khan et al. (34)	Pakistan	Case-control	Yes	NR	160	71	89	18–35^b^		Within 48 h after delivery	≥1 site PPD ≥4 mm (WHO)	LBW	3.17 (1.43–7.05)^†^	PS is a significant risk factor for LBW
Kumar et al. (35)	India	Prospective cohort	/	/	340	208	132	18–35^b^	18–31^b^	14–20 weeks of gestation	CAL and PPD ≥4 mm in ≥1 sites	PTB LBW SGA	1.49 (0.71–3.14)^†^ 1.90 (1.25–3.79)^†^ 1.45 (0.51**–**4.14)	PS (but not GS) is associated with adverse pregnancy outcomes
Macedo et al. (36)	Brazil	Case-control	Yes	No	296	46	250	18–40^b^		Within 48 h after delivery	≥4 teeth with ≥1 sites with PPD ≥4 mm and CAL ≥3 mm at the same site (Lopez)	PTB	1.62 (0.80–3.29)^†^	PD is not associated with PTB
Mathew et al. (37)	India	Case-control	Yes	Yes	160	11	149	18–35^b^		NA	≥1 site PPD ≥4 mm and CAL ≥2 mm	LBW	4.94 (1.03–23.65)*	PD is associated with LBW
Micu et al. (38)	Romania	Case-control	No	Yes	194	38	156	29.1 ± 5.7^a^		Within 72 h after delivery	≥4 teeth with ≥1 sites with PPD ≥4 mm and CAL ≥3 mm at the same site (Lopez)	PTB	2.26 (1.06- 4.82)^†^	PD and its severity might, in part, be considered as contributor to PTB
Moore et al. (39)	UK	Prospective cohort	/	/	546	269	277	32.0 ± 5.1^a^	28.6 ± 5.8^a^	NA	>5 sites with PPD ≥5 mm > 3 sites CAL ≥3 mm	PTB LBW	NA NA	PD is not associated with PTB or LBW in this population
Nabet et al. (40)	France	Case-control	No	Yes	1,955	266	1,689	>18^b^		2–4 days after delivery	≥4 teeth with ≥1 sites with PPD ≥4 mm and CAL ≥ 3 mm at the same site (Lopez)	PTB	1.12 (0.85–1.48)^†^	PS is associated with an increased risk of PTB
Novak et al. (41)	Hungary	Case-control	No	Yes	242	77	165	NA		Within 72 h after delivery	PD ≥4 mm at least at one site and BOP ≥50% of the teeth	PTB LBW	1.95 (1.01–3.74)^†^ 2.58 (1.29–5.16)	PS might be a triggering factor and can be associated with PTB and LBW
Offenbacher et al. (42)	USA	Prospective cohort	/	Yes	1,020	735	285	28.2 ± 6.6^a^		1st or 2nd prenatal visit	PD ≥3 or CAL ≥2	PTB	1.2 (0.9–1.7)^†^	PD increases relative risk for PTB
Pitiphat et al. (43)	USA	Prospective cohort	/	/	1,635	62	1,573	35.2 ± 3.9^a^	35.2 ± 3.9^a^	2nd trimester of pregnancy	Radiography: ≥1 site with bone loss of ≥3 mm	PTB SGA	1.74 (0.65–4.66)^†^ 2.11 (0.76–5.86)	PS is an independent risk factor for poor pregnancy outcome among middle-class women
Ryu et al. (44)	Korea	Case-control	Yes	Yes	172	61	111	19–43^b^		2–5 days after delivery	≥2 teeth CAL >3.5 mm (CDC-APP)	PTB	1.50 (0.74–3.03)^†^	PD showed no association with PTB
Saddki et al. (45)	Malaysia	Prospective cohort	/	/	472	232	240	NA		2nd trimester of pregnancy	≥4 sites with PPD ≥4 mm, and CAL ≥3 mm at the same site, with BOP	LBW	3.84 (1.34–11.05)^†^	PS increases risk of LBW
Souza et al. (46)	Brazil	Case-control	No	Yes	951	163	788	NA		NA	≥4 sites with PPD ≥4 mm, and CAL ≥3 mm at the same site, with BOP	LBW	1.00 (0.61–1.68)^†^	PD is not associated with LBW
Tejada et al. (47)	Switzerland	Case-control	Yes	Yes	429	125	304	≥18^b^		Within 24–72 h after delivery	≥ 2 interproximal sites with CAL ≥4 mm, not on the same tooth (CDC-APP)	PTB	2.38 (1.36–4.14)^†^	PTB is associated with PS
Turton et al. (48)	South Africa	Prospective cohort	/	/	443	320	123	24.13 ± 5.30^a^		NA	PD ≥3 mm or CAL ≥2 mm (Offenbacher)	PTB LBW	NA NA	PD is a risk indicator for adverse pregnancy outcomes
Vogt et al. (49)	Brazil	Prospective cohort	/	/	327	156	171	18–42^b^		Before 32 weeks of gestation	≥4 teeth showing ≥1 site with 4 mm of PPD and CAL at the same site, with BOP	PTB LBW SGA	3.47 (1.62–7.43)^†^ 2.93 (1.36–6.34)^†^ 2.38 (0.93–6.10)*	PD was a risk factor for PTB, LBW among Brazilian low risk pregnant women.

*PTB, preterm birth; LBW, low birth weight; SGA, small for gestational age; PD, periodontal disease; PS, periodontitis; GS, gingivitis; NA, not available*.

*^a^Data as mean ± SD*.

*^b^Data as range*.

*^†^Adjusted odds ratio*.

*Crude odds ratio.

### Methodological Quality

Results of quality assessment of the included studies using NOS for observational studies are presented in Table 2. All the included studies scored at least one star in each of the three categories: the selection and comparability of the study groups and confirmation of the outcome of interest. Overall, 10 studies were graded as high quality, and 14 studies were recognized as low quality due to an NOS score of <7. Management of possible confounders of each study is presented in Supplementary Table 2. Notably, 7 studies recruited subjects matching for confounders such as age and birth order. In 11 studies, the history of periodontal treatment was excluded before recruitment. As an important confounder of adverse neonatal outcomes, age was adjusted in 13 studies. As another important confounder, genitourinary tract infection (GTI) was adjusted in 7 studies. Management of other factors such as parity, smoking, and drug abuse in each study is recorded in Supplementary Table 2.

**Table 2:** Assessment of risk of bias based on the Newcastle–Ottawa Scale.

**Author**	**Selection**	**Comparability^***a***^**	**Exposure/ outcome**	**Total**
Agueda et al. (26)	****	*	***	8
Baskaradoss et al. (27)	**	**	**	6
Bassani et al. (28)	**	**	**	6
Boggess et al. (29)	***	*	***	7
Cruz et al. (30)	**	*	**	5
Erchick et al. (31)	***	*	**	6
Gomes-Filho et al. (32)	**	*	**	5
Jacob and Nath (33)	**	*	**	6
Khan et al. (34)	***	*	***	7
Kumar et al. (35)	***	**	***	8
Macedo et al. (36)	**	**	**	6
Mathew et al. (37)	**	**	**	6
Micu et al. (38)	***	**	**	7
Moore et al. (30)	***	*	**	6
Nabet et al. (40)	**	**	**	6
Novák et al. (41)	***	*	***	7
Offenbacher et al. (42)	***	**	***	8
Pitiphat et al. (43)	**	**	**	6
Ryu et al. (44)	***	*	**	6
Saddki et al. (45)	****	*	***	8
Souza et al. (46)	***	**	**	7
Tejada et al. (47)	**	**	**	6
Turton et al. (48)	****	*	**	7
Vogt et al. (49)	**	**	**	6

^a^Except for comparability which can be awarded a maximum of two stars, other items were given a maximum of one star for each stud. *Score of corresponding questions.

### Comparison in APOs

**Figure 2:**
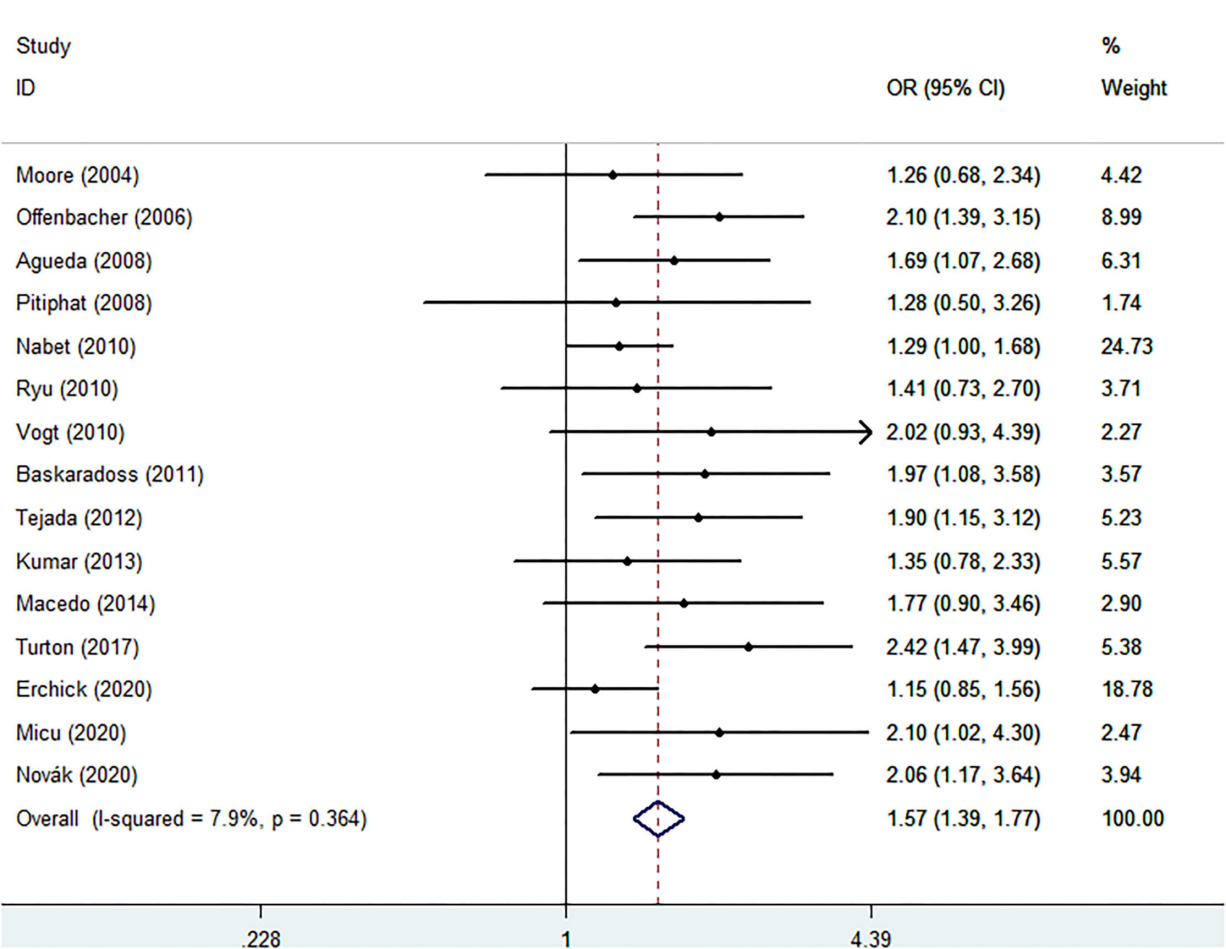
Forest plot of preterm birth events.

**Figure 3:**
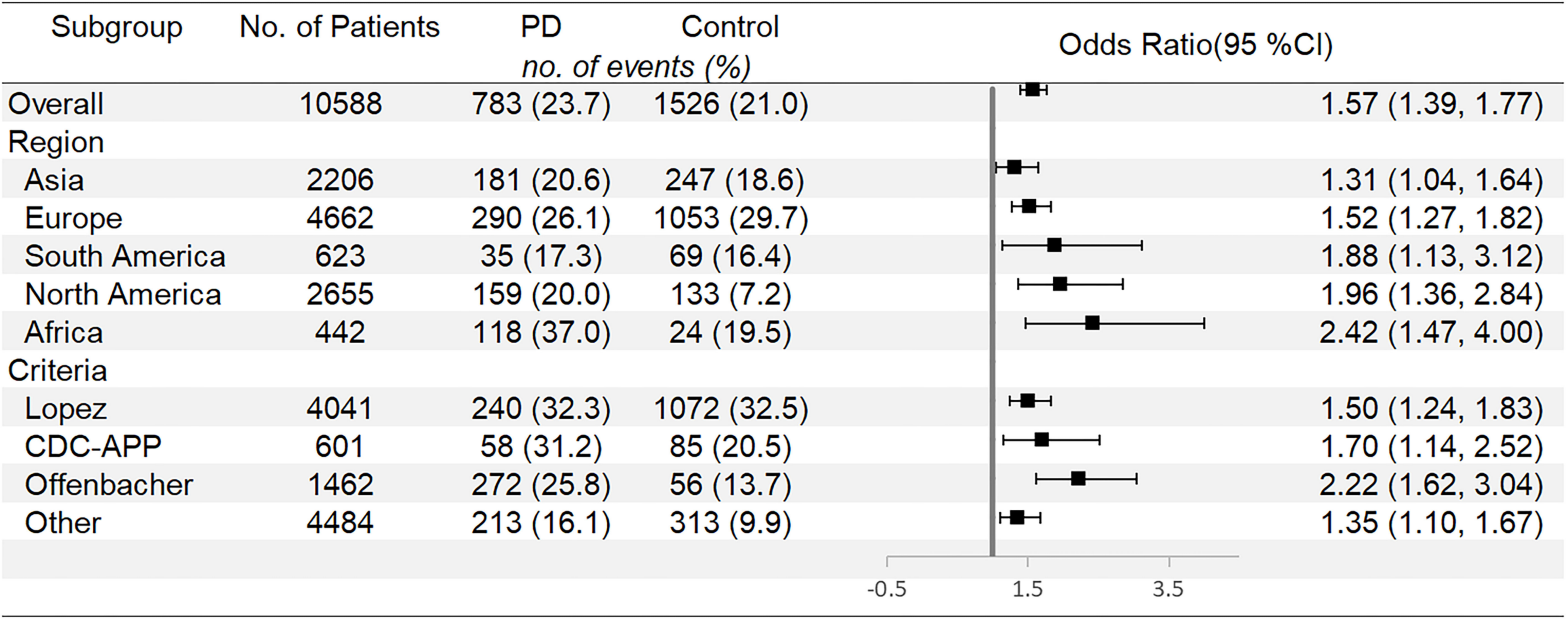
Subgroup analyses for preterm birth.

According to the meta-analysis presented in Figure 2, increased risk of PTB (OR: 1.57, 95% CI: 1.39–1.77, *P* < 0.00001, Ph = 0.364; *I*^2^ = 7.9%) was found in women with PD. Subgroups with increased risks of PTB are listed in Figure 3. In subgroup analyses by region, women with PD in Africa have a higher risk of PTB compared with other regions, and the pooled OR appears significant (OR: 2.42, 95% CI: 1.47–4.00), while Asian women with PD show a relatively lower risk (OR: 1.31, 95% CI: 1.04–1.64). Subgroup analysis showed that different criteria of PD show a significantly different risk of PTB, using criteria operated by Offenbacher, the OR of PTB increased to 2.22 (95% CI: 1.62–3.04); however, Lopez's criteria showed a relatively conservative risk (OR: 1.50, 95% CI: 1.24–1.83).

**Figure 4:**
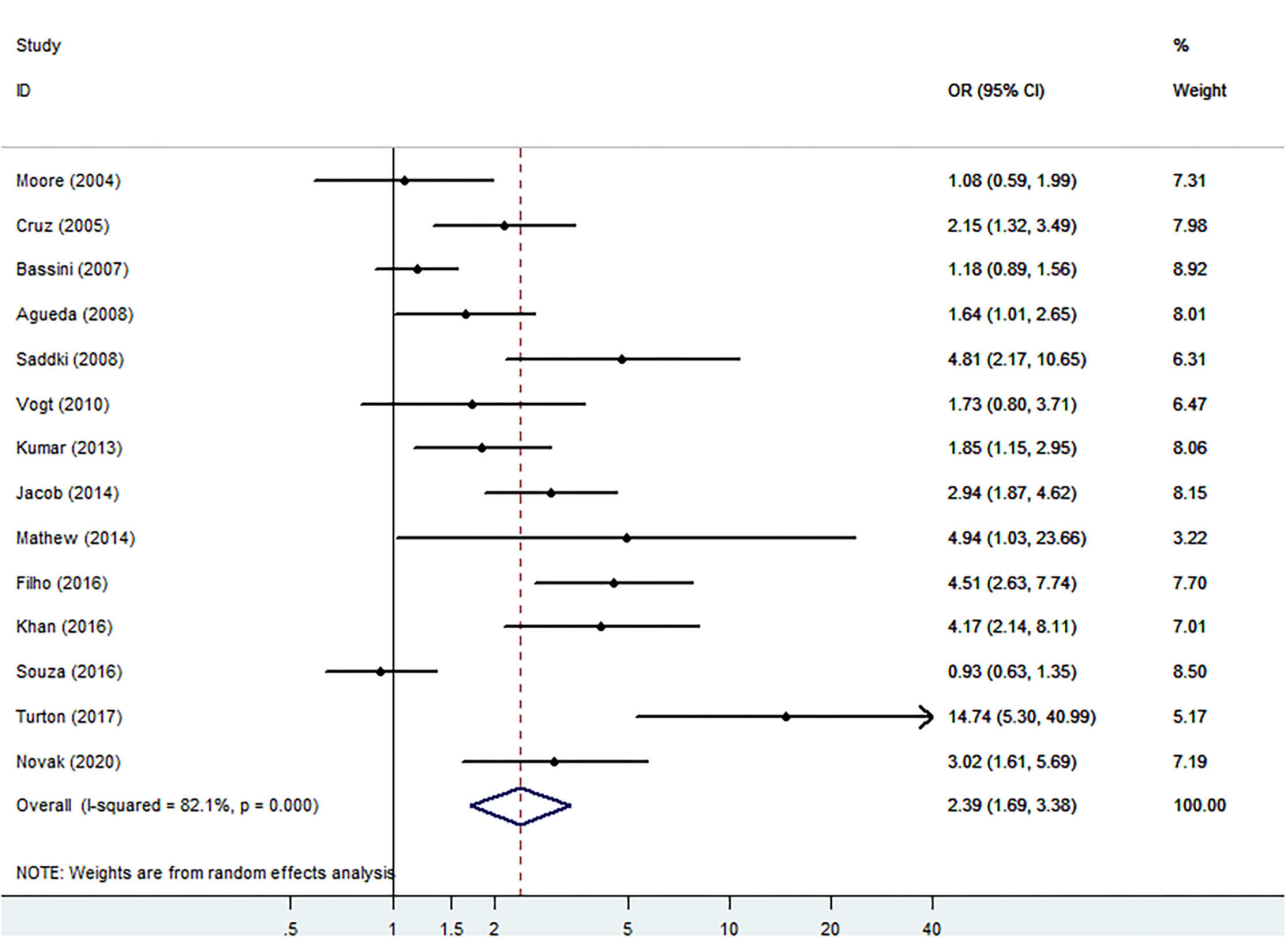
Forest plot of low birth weight (LBW) events.

**Figure 5:**
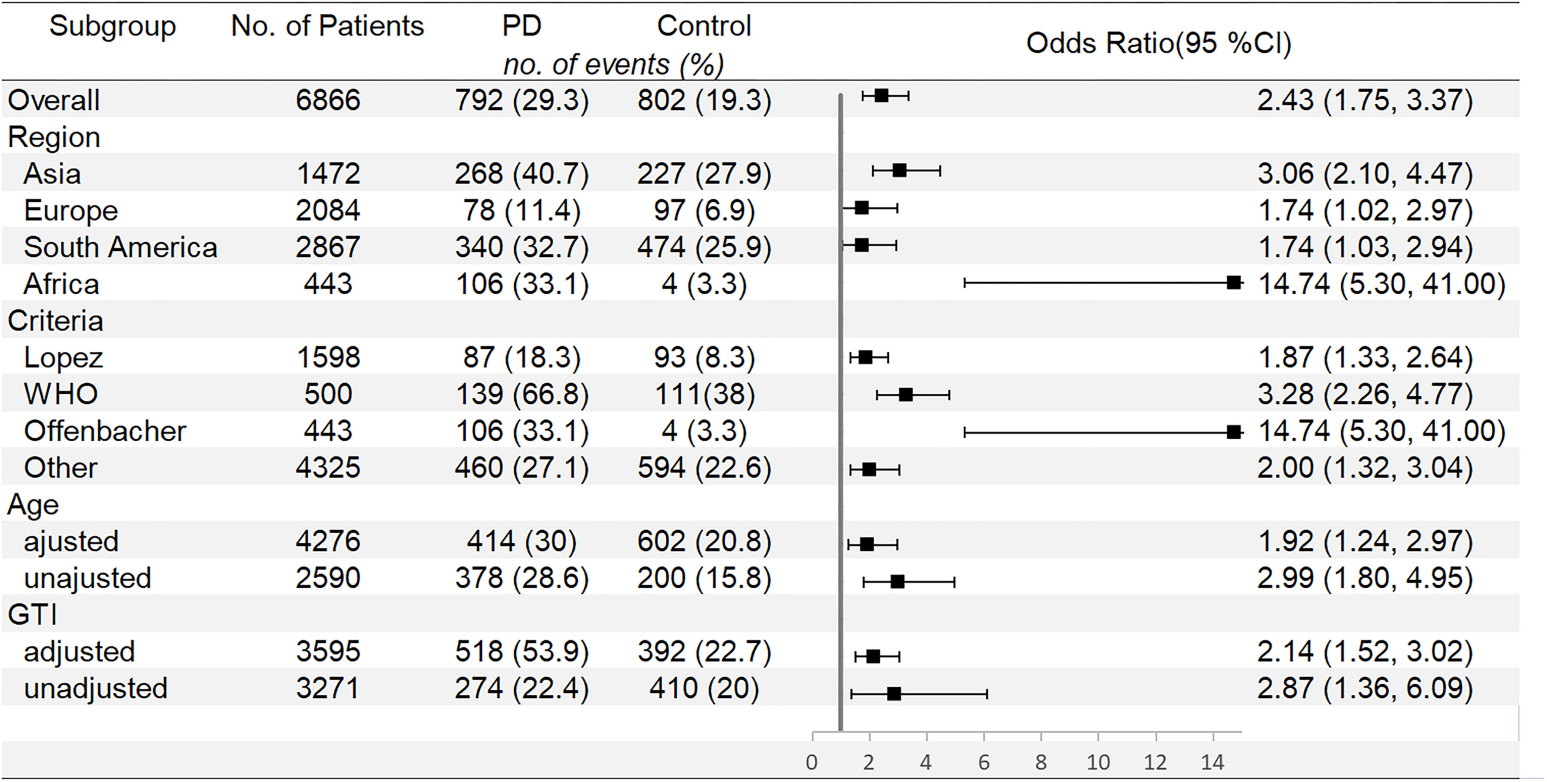
Subgroup analyses for LBW.

Meta-analysis of LBW is shown in Figure 4. Women with PD also shows increased risk of LBW compared with periodontal healthy women (OR: 2.43, 95% CI 1.75–3.37, *P* < 0.00001, Ph = 0.000; *I*^2^ = 82.1%). Figure 5 shows subgroup analyses for LBW. When it comes to LBW, Asia women with PD have the highest risk of LBW (OR: 3.06, 95% CI: 2.10–4.47). Different criteria of PD also show different risks of LBW. Offenbacher's criteria present the highest risk of LBW (OR: 14.74, 95% CI: 5.30–41.00). When the subgroup is divided by whether or not age is adjusted, studies with unadjusted age pooled higher OR (2.99, 95% CI: 1.80–4.95) compared with studies with adjusted age (OR: 1.92, 95% CI: 1.24–2.97). The same result is found according to GTI, with unadjusted studies pooled OR significantly higher than adjusted studies (OR: 2.87, 95% CI: 1.36–6.09 to OR: 2.14, 95% CI: 1.52–3.02).

**Figure 6:**
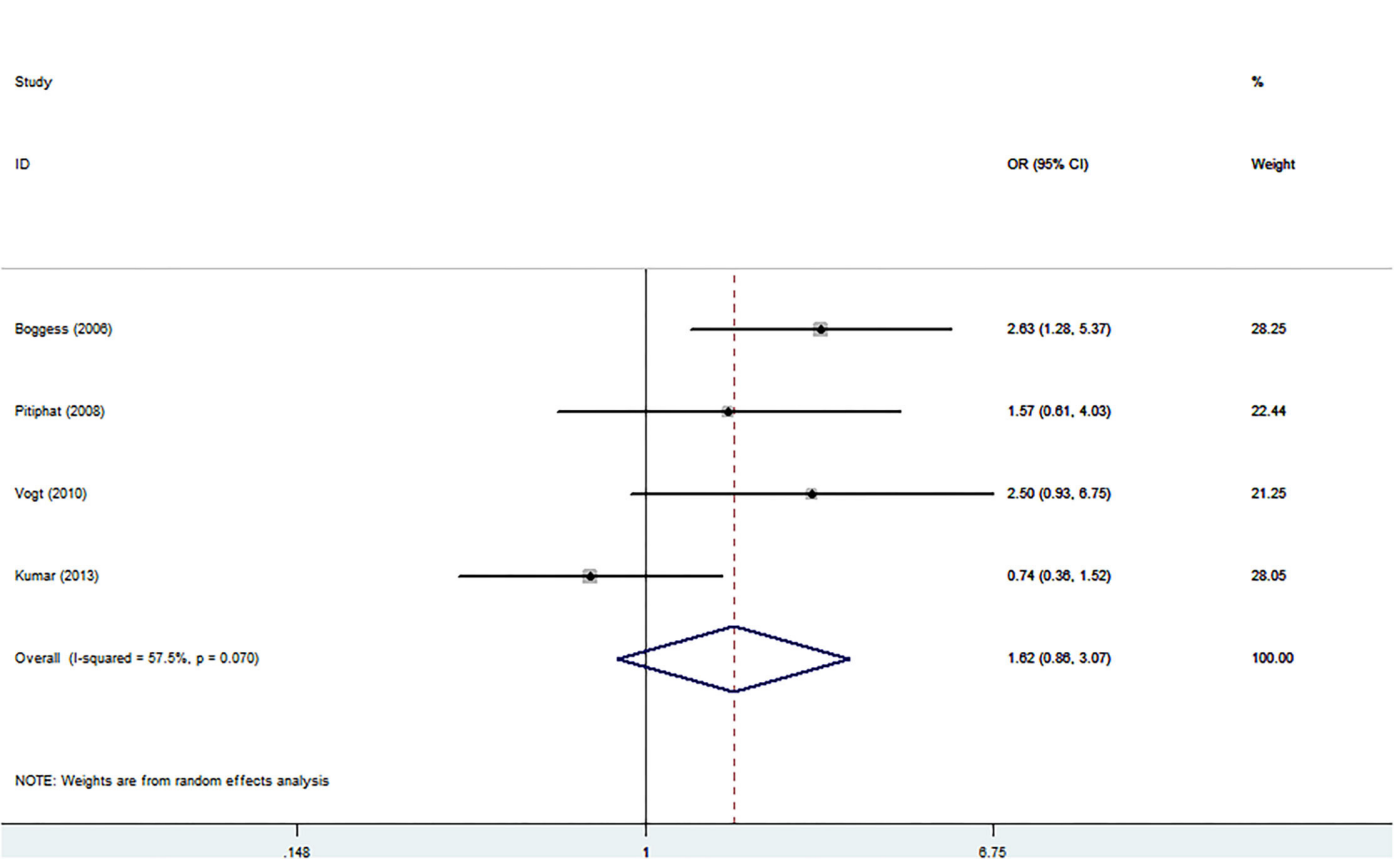
Forest plot of small for gestational age events.

However, OR was 1.62 (95% CI: 0.86–3.07, *P* = 0.136, Ph = 0.07, *I*^2^ = 57.5%) for SGA presented in Figure 6 and illustrate no significant association between PD and SGA. The detailed results stratified by study characteristics are presented in Supplementary Tables 3–6.

### Sensitivity Analysis and Publication Bias

Sensitivity analyses and publication bias of each outcome are shown in Supplementary Table 7. The sensitivity analysis reveals an influence on the pooled OR and 95% CI of SGA when Kumar's study was omitted (OR: 2.25, 95% CI: 1.37–3.69), while PTB and LBW show less influence. Publication bias was detected by Egger's linear regression test in each outcome, and only SGA shows no publication bias.

## Discussion

Our meta-analysis showed that pregnant women with PD had a 1.57-fold higher risk of occurring PTB and 2.43-fold higher risk of delivering LBW infants than pregnant women with a healthy periodontium. Maternal PD is an important risk factor of PTB and LBW. Moreover, women from different regions faced different risks, owing to different genetic backgrounds, dietary habits, oral hygiene habits, and preventive healthcare policies among regions. In addition, we also found that studies without adjustment of underlying confounders such as age and UTI will magnify the effect of PD on adverse neonatal outcomes.

There were numerous studies reminding vaginal infection as an important factor in adverse neonatal outcomes (50, 51). However, some studies pointed out that low-level persistent inflammation rather than infection may trigger PTB too (7, 52). Maternal PD was a chronic exposure to oral Gram-negative pathogens, which was a preventable and treatable risk factor for adverse neonatal outcomes. In women with PD, periodontal pathogens gained access to the blood circulation, and they could reside in the placenta and, worst of all, penetrate the placental barrier into the amniotic fluid and fetal circulation. Through the examination of the placental samples from women with PTB, studies had identified several microorganisms which were associated with PD, such as *Aggregatibacter actinomycetemcomitans, Fusobacterium nucleatum, Porphyromonas gingivalis*, and *Prevotella intermedia*, while these microorganisms were absent from placenta from women without periodontal infection (53, 54). The presence of these substances in the fetal-placental section could stimulate the fetal immune and inflammatory response, accompanied by the production of IgM antibodies and the increased secretion of inflammatory mediators, which in turn may cause PTB (55, 56). In this case, LBW was a result of PTB. Moreover, chronic inflammation may cause structural changes in the placenta, leading to insufficient fetal nutrient support and also resulting in LBW (57, 58).

Another explanation was that in these women, PD elevated the local and systemic level of inflammatory cytokines, promoting trophoblasts and chorioamnionic cells to secret interleukins (e.g., IL-1), prostaglandins (e.g., PGE2), tumor necrosis factor-α (TNF-α), and matrix metalloproteinases (MMPs) (59, 60). IL-1 and TNF-α acted as initial proinflammatory mediators and directly enhanced PGE2 production by inducing cyclooxygenase-2 (COX-2) expression both in the amnion and the decidua. Meanwhile, the production of MMPs in the amnion chorion, decidua, and cervix would also be enhanced, which would degrade the extracellular matrix of the fetal membranes and cervix (58, 61). The above process contributes to adverse neonatal outcomes.

This study was based on a larger and updated database, with more observed outcomes and detailed subgroup analysis compared with a previous similar study (62–64). The previous meta-analysis on PD and pregnancy outcomes showed high heterogeneity (65, 66), and the effect of various PD criteria and management of confounders may explain this high degree of heterogeneity. Our study distributes the studies into several subgroups according to different criteria, explaining the source of heterogeneity in LBW to some extent. In addition, subgroup analyses of whether adjusted GTI can eliminate the heterogeneity in SGA (Supplementary Table 7) illustrated the importance of management of confounders in future studies. The limitation should be considered when interpreting the results of this study. Egger's test revealed an apparent bias that suggested the presence of a potential publication bias, inflated estimates by a flawed methodological design in smaller studies, and/or a lack of publication of small studies with opposite results. In addition, since only PD was studied, it is unknown whether other oral diseases such as an ulcer or dental caries also had adverse effects on pregnancy outcomes. Finally, the treatment and intervention of the subjects during pregnancy had not been recorded in most literature, which makes it impossible to analyze the impact of pregnancy intervention on adverse neonatal outcomes.

Our study has potential implications for current clinical practice. Since periodontal status is a modifiable risk factor of adverse neonatal outcomes, we suggested that either obstetricians or dentists should raise their awareness of the periodontal condition in children-bearing women, and these patients should be educated to take special care of their periodontal health, provided with antenatal dental checkups and actively treated once they have signs of periodontal infection. These will reduce the incidence of adverse neonatal outcomes in pregnant women to some extent. American Academy of Periodontology (AAP) recommends bringing PD control into preconception programs and treating the disease during pregnancy, although many pregnant women are worried about the adverse effects of treatment during pregnancy, periodontal treatment is not a risk for pregnancy, and the benefits outweigh the risks of ignorance (67). Large randomized controlled trials that control the therapeutic measures need to be investigated.

In conclusion, PD was suggested as a risk factor for PTB and LBW, with a severer adverse effect in Africa and Asia separately. Therefore, improving oral health should be emphasized in children-bearing women, for the purpose of reducing the incidence of adverse neonatal outcomes. It is necessary to add oral examination into the pre-pregnancy evaluation. If PD was diagnosed, a previous treatment was suggested to avoid its adverse effect on obstetric outcomes.

## Data Availability Statement

The raw data supporting the conclusions of this article will be made available by the authors, without undue reservation.

## Author Contributions

YZ performed study selection, full-text review, and drafted the manuscript. WF performed database searches. JL helped with data analyses and provided useful comments on outcome assessment. LC performed study selection, full-text review, and the revision of the manuscript. Z-JC contributed to the study concept and design. All authors contributed to this study and approved the submitted version.

## Funding

This study was supported by the National Key Research and Development Program of China (2018YFC1004301), Shandong Provincial Key Research and Development Program (2018YFJH0504), Natural Science Foundation of Shandong Province of China (ZR2020MH065), and Taishan Scholars Program for Young Experts of Shandong Province, and China Health Promotion Foundation (association between ovarian response and embryo quality in women with normal ovarian reserve undergoing *in vitro* fertilization using a standard long protocol: a retrospective cohort study; association between the numbers of retrieved oocytes and pregnancy outcomes in women with normal ovarian reserve undergoing *in vitro* fertilization: a retrospective cohort study). Funders did not have any role in study design, data collection, and data analyses.

## Conflict of Interest

The authors declare that the research was conducted in the absence of any commercial or financial relationships that could be construed as a potential conflict of interest.

## Publisher's Note

All claims expressed in this article are solely those of the authors and do not necessarily represent those of their affiliated organizations, or those of the publisher, the editors and the reviewers. Any product that may be evaluated in this article, or claim that may be made by its manufacturer, is not guaranteed or endorsed by the publisher.

## Abbreviations

PD: periodontal disease
PTB: preterm birth
LBW: low birth weight
SGA: small for gestational age
OR: odds ratio
CIs: confidence intervals
*I* ^2^: heterogeneity
GTI: genitourinary tract infection
PGE2: prostaglandins
TNF-α: tumor necrosis factor-α
MMPs: matrix metalloproteinases
COX-2: cyclooxygenase-2.
