# Supplementary material for: Periodontal Disease and Adverse Neonatal Outcomes: A Systematic Review and Meta-Analysis
Source: Front Pediatr. 2022 May 4;10:799740. doi: 10.3389/fped.2022.799740 (PMC9114501; doi:10.3389/fped.2022.799740)
Supplement: Supplementary file 1 [file Data_Sheet_1.pdf]

Supplemental Tables

Supplemental Table 1: Search strategy.

Supplemental Table 2: Management of possible confounders.

Supplemental Table 3: Factors for subgroups analysis.

Supplemental Table 4: Subgroup analyses for PTB.

Supplemental Table 5: Subgroup analyses for LBW.

Supplemental Table 6: Subgroup analyses for SGA.

Supplemental Table 7: Sensitive analyses and publication bias for APOs.

Supplemental Table 1: Search strategy.

#1	TS=("Oral Health [mh]" OR "Oral Disease" OR "Periodontal Diseases [mh]" OR "PD" OR "Periodontitis [mh]" OR "Periodontitides" OR "Gingivitis [mh]" OR "Gingivitides" OR "Gingival Disease" OR "Gingivosis")
#2	TS=("Pregnancy Outcome [mh]" OR "Obstetrical Disorder" OR "Obstetrical Diseases" OR "Premature Birth [mh]" OR "Infant, Premature" OR "Preterm Birth" OR "PTB" OR "Premature Delivery" OR "Preterm Delivery" OR "PTD" OR "Infant, Low Birth Weight [mh]" OR "Low Birth Weight" OR "LBW" OR "Infant, Small for Gestational Age [mh]" OR "Small for Gestational Age" OR "SGA")
#1 AND #2	

Unless otherwise declared, terms used in search strategy were free text terms.

TS, topic.

Supplemental Table 2: Management of possible confounders.

**Author/Year**	**Age**	**BMI**	**Ethnicity**	**Parity**	**Smoking**	**Alcohol**	**Drug abuse**	**History of events[a]**	**Pregenancy Complication[b]**	**Genitourinary tract infection[c]**	**Marital status**	**Education**	**Socioeconomic**	**Prenatal care**	**PD treatment**
Agueda *et al.* 2008	A	N	A	NR	A	N	E	A	A	A	NR	N	N	A	NR
Baskaradoss *et al.* 2011	A	N	NR	N	N	NR	NR	A	N	A	NR	N	N	N	E
Bassini *et al.* 2007	A	NR	N	N	A	N	NR	A	A	A	NR	N	N	A	NR
Boggess *et al.* 2006	A	N	N	NR	A	N	A	N	A	N	A	NR	N	NR	NR
Cruz *et al.* 2005	N	NR	NR	NR	N	N	NR	NR	NR	A	N	N	N	N	E
Erchick *et al.* 2020	N	N	N	N	N	NR	NR	NR	NR	N	E	N	N	NR	NR
Filho *et al.* 2016	A	A	N	A	N	N	NR	N	NR	N	NR	NR	A	A	E
Jacob *et al.* 2014	N	NR	NR	E	A	NR	NR	NR	A	A	NR	N	A	A	E
Khan *et al.* 2016	NR	NR	NR	E	N	NR	NR	NR	E	E	NR	A	A	N	E
Kumar *et al.* 2013	A	A	NR	E	NR	NR	NR	NR	N	E	NR	A	A	NR	E
Macedo *et al.* 2014	N	NR	N	N	E	E	E	N	NR	N	N	N	N	N	N
Mathew *et al.* 2014	A	NR	NR	N	N	NR	NR	N	E	N	A	N	N	N	E
Micu *et al.* 2020	N	N	NR	N	A	NR	NR	N	E	E	NR	N	N	N	E
Moore *et al.* 2004	A	NR	N	N	N	N	NR	N	N	N	NR	NR	N	NR	N
Nabet *et al.* 2010	A	A	A	A	A	NR	N	NR	N	NR	A	A	N	N	NR
Novak *et al.* 2020	N	NR	NR	N	A	NR	NR	NR	E	E	NR	A	A	NR	E
Offenbacher *et al.* 2006	A	NR	A	A	A	N	N	A	A	N	A	NR	A	NR	NR
Pitiphat *et al.* 2008	A	A	A	A	A	N	NR	A	NR	A	N	N	A	NR	NR
Ryu *et al.* 2010	A	NR	NR	A	A	A	NR	A	NR	NR	NR	NR	NR	NR	N

Saddki *et al.* 2008	N	N	NR	N	N	E	NR	N	NR	NR	NR	A	N	N	E
Souza *et al.* 2016	A	A	N	A	A	NR	NR	N	A	N	N	A	N	A	E
Tejada *et al.* 2012	NR	NR	NR	N	N	NR	NR	N	E	A	A	NR	A	N	N
Turton *et al.* 2017	N	NR	N	NR	E	E	NR	NR	NR	NR	NR	N	NR	NR	NR
Vogt *et al.* 2010	N	N	N	N	N	N	NR	E	NR	N	N	N	NR	A	NR

A: adjusted confounder; N: unadjusted confounder; NR: not reported; E: confounder as excluded criteria; BMI: Body Mass Index;

[a] whether recorded early events of PTB and/or LBW and/or SGA

[b] whether recorded pregnancy copmplication such as preeclampsia and/or pregnancy-induced hypertension and/or gestational diabetes mellitus

[c] whether recorded genitourinary tract infection and/or chorioamnionitis

Supplemental Table 3: Factors for subgroups analysis.

Outcomes	Region	Design	Criteria	NOS quality	Adjusted Age	Adjusted GTI
PTB	Asia<br>Europe<br>South America<br>North America<br>Africa	Prospective cohort<br>Case-control	Lopez<br>CDC-APP<br>Offenbacher<br>Other	High<br>Low	Yes<br>No	Yes<br>No
LBW	Asia<br>Europe<br>South America<br>Africa	Prospective cohort<br>Case-control	Lopez<br>WHO<br>Offenbacher<br>Other	High<br>Low	Yes<br>No	Yes<br>No
SGA	Asia<br>South America<br>North America	NA	WHO<br>Other	High<br>Low	Yes<br>No	Yes<br>No

NS: No significant result was observed in any subgroup;

NA: Subgroup analyses were not available;

Supplemental Table 4: Subgroup analyses for PTB.

	No. of studies	OR (95% CI)	*P*	Weight (%)	Q	*Ph*	I^2 (%)
**All studies**	15	1.57 [1.39, 1.77]	0.000	100	15.20	0.364	7.9
**Region**							
Asia	4	1.31 [1.04, 1.64]	0.021	31.63	2.52	0.472	0.0
Europe	6	1.52 [1.27, 1.82]	0.000	47.10	4.67	0.457	0.0
South America	2	1.88 [1.13, 3.12]	0.015	5.17	0.06	0.799	0.0
North America	2	1.96 [1.36, 2.84]	0.000	10.72	0.9	0.342	0.0
Africa	1	2.42 [1.47, 4.00]	0.001	5.38			
**Design**							
Prospective cohort	8	1.57 [1.33, 1.86]	0.000	53.45	10.27	0.174	31.8
Case-control	7	1.56 [1.30, 1.87]	0.000	46.55	4.95	0.551	0.0
**Criteria**							
Lopez	5	1.50 [1.24, 1.83]	0.000	39.97	3.33	0.504	0.0
CDC-APP	2	1.70 [1.14, 2.52]	0.009	8.94	0.51	0.475	0.0
Offenbacher	2	2.22 [1.62, 3.04]	0.000	14.37	0.19	0.661	0.0
Other	6	1.35 [1.10, 1.67]	0.005	36.72	4.29	0.509	0.0
**NOS quality**							
High	6	1.94 [1.58, 2.39]	0.000	32.65	3.01	0.698	0.0
Low	9	1.39 [1.19, 1.62]	0.000	67.35	6.06	0.641	0.0

**Adjusted age**							
Yes	8	1.51 [1.28, 1.77]	0.000	59.03	5.44	0.606	0.0
No	7	1.65 [1.37, 1.99]	0.000	40.97	9.26	0.160	35.2
**Adjusted GTI**							
Yes	6	1.86 [1.47, 2.35]	0.000	23.26	1.06	0.958	0.0
No	9	1.48 [1.28, 1.71]	0.000	76.74	11.41	0.180	29.9

GTI: genitourinary tract infection

Supplemental Table 5: Subgroup analyses for LBW.

	No. of studies	OR (95% CI)	*P*	Weight (%)	Q	*Ph*	I^2 (%)
**All studies**	14	2.43 [1.75, 3.37]	0.0000	100.00	72.50	0.000	82.1
**Region**							
Asia	5	3.06 [2.10, 4.47]	0.000	32.75	6.71	0.152	40.3
Europe	3	1.74 [1.02, 2.97]	0.044	22.51	5.30	0.171	62.2
South America	5	1.74 [1.03, 2.94]	0.038	39.57	27.27	0.000	85.3
Africa	1	14.74 [5.30, 41.00]	0.000	5.17			
**Design**							
Prospective cohort	6	2.48 [1.37, 4.51]	0.003	41.33	25.70	0.000	80.5
Case-control	8	2.37 [1.49, 3.76]	0.000	58.67	47.26	0.000	85.2
**Criteria**							
Lopez	2	1.87 [1.33, 2.64]	0.000	15.99	0.60	0.440	0.0
WHO	2	3.28 [2.26, 4.77]	0.000	15.16	0.72	0.397	0.0
Offenbacher	1	14.74 [5.30, 41.00]	0.001	5.17			
Other	9	2.00 [1.32, 3.04]	0.000	63.68	42.02	0.000	81.0
**NOS quality**							
High	7	2.80 [1.56, 5.04]	0.001	50.25	43.34	0.000	86.2
Low	7	2.10 [1.34, 3.30]	0.001	49.75	29.37	0.000	79.6
**Adjusted age**							

Yes	7	1.92 [1.24, 2.97]	0.003	51.60	32.90	0.000	81.8
No	7	2.99 [1.80, 4.95]	0.000	48.40	25.91	0.000	76.8
**Adjusted GTI**							
Yes	7	2.14 [1.52, 3.02]	0.000	55.32	22.75	0.001	73.6
No	7	2.87 [1.36, 6.09]	0.006	44.68	50.36	0.000	88.1

GTI: genitourinary tract infection

Supplemental Table 6: Subgroup analyses for SGA.

	No. of studies	OR (95% CI)	*P*	Weight (%)	Q	*Ph*	I^2 (%)
**All studies**	4	1.62 [0.86, 3.07]	0.136	100	7.06	0.07	57.5
**Region**							
Asia	1	0.74 [0.36, 1.52]	0.412	28.05			
South America	1	2.50 [0.93, 6.75]	0.070	21.25			
North America	2	2.18 [1.23, 3.85]	0.007	50.69	0.75	0.388	0.0
**Criteria**							
WHO	1	2.63 [1.28, 5.37]	0.008	28.25			
Other	3	1.34 [0.65, 2.78]	0.434	71.75	4.13	0.127	51.6
**NOS quality**							
High	2	1.39 [0.40, 4.88]	0.603	56.30	6.07	0.014	83.5
Low	2	1.96 [0.99, 3.88]	0.054	43.70	0.45	0.505	0.0
**Adjusted age**							
Yes	3	1.45 [0.65, 3.19]	0.362	78.75	6.09	0.048	67.1
No	1	2.50 [0.93, 6.75]	0.070	21.25			
**Adjusted GTI**							
Yes	2	1.01 [0.49, 2.11]	0.971	50.50	1.57	0.210	36.3
No	2	2.58 [1.44, 4.62]	0.001	49.50	0.01	0.937	0.0

GTI: genitourinary tract infection

Supplemental Table 7: Sensitive analyses and publication bias for adverse pregnancy outcomes

Outcome	No. of Studies	Effects Model	Variations in Sensitivity Analyses OR (95%CI)	Egger's Test for Publication Bias (*P*)	Variations in Trim and Filled Analyses OR (95%CI)
PTB	15	fixed	NS	0.063	-
LBW	14	random	NS	0.006	-
SGA	4	random	NS	0.699	-

NS: No significant variations introduce
